# Polymeric Nanocomposites for Environmental and Industrial Applications

**DOI:** 10.3390/ijms23031023

**Published:** 2022-01-18

**Authors:** Mohamed S. A. Darwish, Mohamed H. Mostafa, Laila M. Al-Harbi

**Affiliations:** 1 Egyptian Petroleum Research Institute, 1 Ahmed El-Zomor Street, El Zohour Region, Nasr City, Cairo 11727, Egypt; m.hassan@epri.sci.eg; 2 Chemistry Department, Faculty of Science, King Abdul-Aziz University, P.O. Box 80203, Jeddah 21589, Saudi Arabia; lalhrbi@kau.edu.sa

**Keywords:** polymeric nanocomposites, sensing, electromagnetic shielding, water treatment, food packaging

## Abstract

Polymeric nanocomposites (PNC) have an outstanding potential for various applications as the integrated structure of the PNCs exhibits properties that none of its component materials individually possess. Moreover, it is possible to fabricate PNCs into desired shapes and sizes, which would enable controlling their properties, such as their surface area, magnetic behavior, optical properties, and catalytic activity. The low cost and light weight of PNCs have further contributed to their potential in various environmental and industrial applications. Stimuli-responsive nanocomposites are a subgroup of PNCs having a minimum of one promising chemical and physical property that may be controlled by or follow a stimulus response. Such outstanding properties and behaviors have extended the scope of application of these nanocomposites. The present review discusses the various methods of preparation available for PNCs, including in situ synthesis, solution mixing, melt blending, and electrospinning. In addition, various environmental and industrial applications of PNCs, including those in the fields of water treatment, electromagnetic shielding in aerospace applications, sensor devices, and food packaging, are outlined.

## 1. Introduction

Polymeric nanocomposites (PNCs) are important materials for industrial as well as research purposes and are used widely in packaging, energy, safety, transportation, electromagnetic shielding, defense systems, sensors, catalysis, and information industry [1,2,3]. PNCs could resolve several problems and daily challenges of the real world, conferring great future potential to these materials. PNCs are designed based on the principle of size and surface area being associated with much higher reactivity [4]. PNCs are hybrid materials composed of polymers as the matrix and nanomaterials as the nanofillers. PNCs exhibit unparalleled multi-functions due to the incorporation of multi-components into an integrated compatible structure, which enables PNCs to be highly applicable in various electronic, magnetic, and optical applications [5,6]. Two types of polymers are available—natural and synthetic. Natural polymers are the ones that occur in nature, from where these may be extracted for use. Natural polymers are often water-based, such as silk, wool, DNA, cellulose, and proteins [7]. Synthetic polymers, on the other hand, include those that are prepared synthetically, such as nylon, polyethylene, polyester, Teflon, and epoxy. Different inorganic nanofillers, including nanoclays, metal-oxide nanoparticles, carbon nanomaterials, and metal nanoparticles, may be incorporated within a polymer matrix to prepare a PNC with improved properties specific to a particular application [8]. The enhanced properties of the fabricated PNC are a consequence of the uniform distribution of nanofillers within the polymer matrix. However, when the nanomaterial fillers aggregate within the polymer matrix due to the Van der Waals forces among the nanoparticles, the effective and desired properties of the fabricated PNC may exhibit a decline [9,10]. This problem could be resolved by the use of nanomaterials with modified/functionalized surfaces, which would result in enhanced dispersion of the nanofillers within the polymer matrix through the enhancement of the reaction and compatibility between the nanofillers and the polymer matrix at their interface [11]. The surface functionalization of the nanofillers may be achieved by fabricating an organic coating through a physical or chemical reaction which would generate a PNC for advanced applications [12,13]. Several applications are reported for such fabricated PNCs in different fields [3,14]. Among these are the stimuli-responsive polymers, or smart polymers as these are alternatively referred to, which exhibit remarkable changes in their properties, in terms of responding to even the slightest changes in the environmental conditions. Stimuli-responsive polymers are sensitive to certain triggers from their external environment, including changes in the temperature, light, electrical field, magnetic field, and chemicals. The present review discusses such polymer nanocomposites, including their preparation methods and applications in the fields of water treatment, electromagnetic shielding in aerospace applications, sensor devices, and food packaging, among others.

## 2. Preparation Methods

The preparation of PNCs involves the incorporation of different nanofillers into the polymer matrix [15,16]. PNCs may be fabricated using various techniques, such as *in situ* synthesis, solution mixing, melt processing, electrospinning, etc. The selection of the preparation method depends on various parameters, such as the polymeric system used, the target application field, particle distribution, size, etc. [17,18] The various methods of preparation available for PNCs are discussed below.

### 2.1. In Situ Synthesis

**Figure 1:**
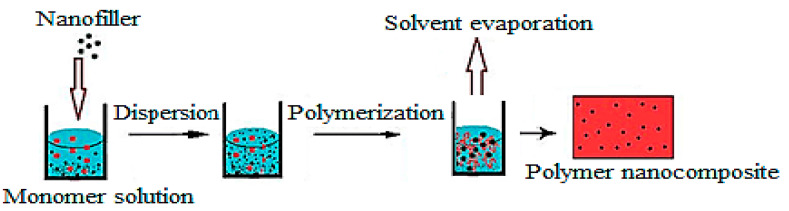
Schematic illustration for the in situ polymerization method (taken from [27]).

The in situ synthesis of PNCs includes several steps. The first step is the synthesis of the nanomaterial in the presence of polymer. The second step, as illustrated in Figure 1, is the synthesis of the PNC through the polymerization of monomers in the presence of the synthesized nanomaterial. The third step is the simultaneous synthesis of both polymer and nanomaterial. The main advantage of the in situ synthesis of PNCs is that it enables achieving high uniform dispersion of nanofillers throughout the polymer matrix, which improves compatibility and enables high interaction at the interface [19,20]. Several types of PNCs with different characteristics may be fabricated using the in situ method. The characteristics of the fabricated material would depend on the nature of the nanoparticle precursor and the polymer used. In the preparation of high-quality PNCs, two challenges are mainly encountered: (i) the dispersion of the nanoparticles within the polymer matrix and (ii) the interaction between the polymer and the nanoparticles at their interface. These challenges may be overcome by controlling several factors, such as using a suitable solvent, the appropriate functionalization route and selective dispersion techniques [21]. 

The in situ synthesis method requires several starting materials, such as the polymer, monomer, precursor, nanoparticles, etc. The additional requirements include glass, heat source, long duration, and high energy for conducting the chemical reactions. While in situ synthesis is a promising method for obtaining PNCs with uniform and well-defined structures, it produces only small amounts of the final product and, therefore, is not suitable for large-scale production [22]. The in situ synthesis of PNCs holds better potential when the nanofillers are functionalized. In this context, the synthesis of poly N-isopropylacrylamide (PNIPAM)/magnetic nanoparticles (MNPs) using the in situ synthesis method has been reported. In that study, the MNPs were prepared through co-precipitation and then coated with a silica shell and subsequently modified with γ-methaacryloxpropyl triisoprooxidesilane. The prepared MNPs were then inserted into the NIPAM monomer via the “grafting-through” reaction, producing the desired nanocomposite [23]. In another study, polymethyl methacrylate (PMMA)/superparamagnetic iron oxide NPs (SPIONs)/PEG bis(amine) nanocomposites were applied to water treatment. In that study, the NPs were fabricated through co-precipitation and subsequently modified with PMMA using the emulsion polymerization process in SPION suspension. This was followed by the insertion of PEG bis(amine) onto the PMMA-coated SPIONs [13]. Munnawar et al. prepared a nanocomposite of chitosan (Ct) and ZnO nanoparticles through chemical precipitation. In the chemical reaction, a coordination bond was formed between the functional groups of Ct and Zn^+2^ ions [24]. In another study, graphene oxide was grafted with polylactic acid (PLA) through in situ polycondensation of L-lactic acid in the presence of graphene oxide to produce the nanocomposite [25]. Similarly, a nanocomposite comprising PLA and cloisite was fabricated through ring-opening polymerization of lactide at various loadings of cloisite with the assistance of microwave heating [26].

### 2.2. Solution Mixing

**Figure 2:**
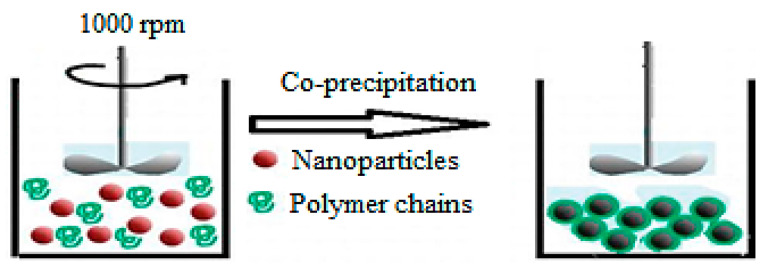
Schematic illustration for the solution mixing method (taken from [27]).

The solution mixing method for preparing PNCs relies on evaporating the solvent from the solution, as illustrated in Figure 2. The polymer is first dissolved in a volatile solvent, following which the nanomaterials are dispersed into the polymer solution using sonication. Afterward, the PNCs are produced by performing rapid solvent evaporation [28]. The solution mixing method is a simple and economic approach that does not require a complex design or a large number of chemicals. Moreover, there are no additional energy requirements, and high amounts of PNCs are produced within a short duration. Several studies have demonstrated the preparation of PNCs using the solution mixing method. For instance, the poly (3-hydroxybutyrate-co-3-hydroxyvalerate)/ZnO nanoparticles nanocomposite was prepared using the solution mixing without involving the use of coupling agents, and the process involved the formation of a hydrogen bond between the polymer and the nanoparticles [29]. The solution mixing method has also been used for the preparation of starch nanocomposite films with clay nanolayers, which exhibited enhanced antibacterial activity toward *S. aureus* and *E. coli* [30]. Furthermore, Ct nanocomposite with TiO_2_ nanoparticles was prepared using the solution mixing method. The mechanism involved in this preparation could be explained based on the pH at the point of zero charge (PZC), a point at which the sum of all negative surface charges balances the sum of all positive charges. When the pH of the solution is below the pH at PZC, the surface of the TiO_2_ nanoparticles becomes positively charged. Conversely, the surface becomes negatively charged when the solution pH is higher than the pH at PZC. In solutions with pH lower than the pH at PZC of TiO_2_ nanoparticles and the pKa of Ct, electrostatic repulsion would occur between the charged surface of the TiO_2_ nanoparticles and the chains of Ct, which would enable the complete stretching of the flexible Ct chains, thereby resulting in the dispersion of TiO_2_ nanoparticles into the Ct matrix [31].

### 2.3. Melt Blending

**Figure 3:**
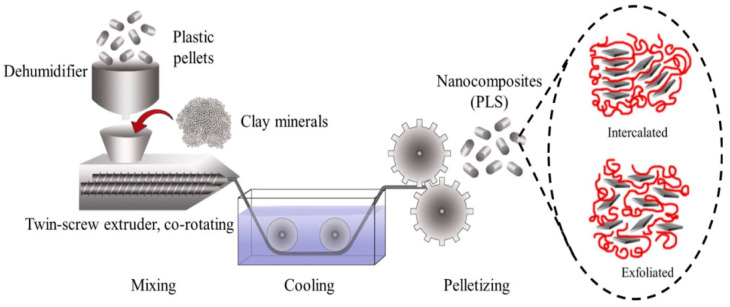
Schematic representation for preparing polymer nanocomposites by melt blending (taken from [37]).

The melt blending method for preparing PNCs involves melting the polymer followed by the dropwise addition of the nanomaterials. Figure 3 presents the schematic representation of PNC preparation using the melt blending process. The factors that affect the melt blending process include the type of polymer used, the type of nanoparticles used, the temperature of the process, and the process duration. The production of PNCs through melt blending is achieved using ordinary compounding devices such as mixers or extruders [32]. In comparison to the in situ synthesis and solution mixing method for PNC production, melt blending has the advantage that it does not involve the use of organic solvents. Melt blending is compatible with the existing industrial processes, such as extrusion and injection molding. This process allows continuous, rapid, and simple transformation of the raw ingredients into the desired product. A high temperature during the melt blending process may result in the thermal degradation of the polymers used. Therefore, it is important to adjust the temperature accordingly and use a system for producing PNCs that has appropriate conditions specific to the desired processing efficiency and the desired shape and properties of the final products [33]. Nanocomposites based on PLA and ZnO nanoparticles were prepared using the melt blending process conducted in a HaakeMiniLab II co-rotating twin-screw extruder at 180 °C, 15 min of retention time, and 20 rpm [34]. In another study, poly(-3-hydroxybutyrate-co-3-hydroxyvalerate) (PHBV) nanocomposites with modified montmorillonite and halloysite were prepared through melt blending using a twin screw-rotating extruder at 80 rpm over the temperature range of 150–165 °C [35]. Darwish et al. reported using melt blending for preparing polypropylene nanocomposites using physically prepared Ct/ZnO nanocomposite. Another study involved performing melt blending in a Brabender mixer at 180 °C and 60 rpm as the temperature and rotor speed, respectively [36].

### 2.4. Electrospinning

**Figure 4:**
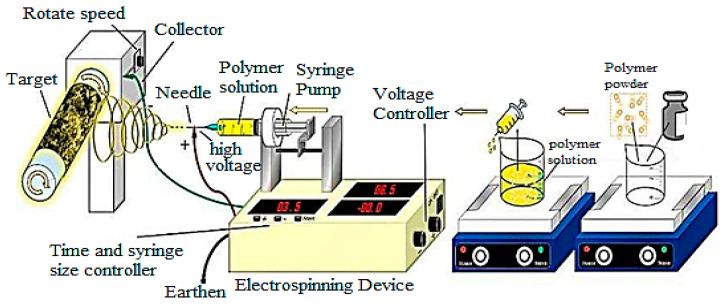
The set-up of electrospinning technique (taken from [45]).

Electrospinning is recognized as a successful method for producing nanofibers. The various factors that affect the electrospinning process include the flow rate, polymer concentration, solution viscosity, air humidity, and electric field intensity [38]. The set-up of the electrospinning technique is depicted in Figure 4. Electrospinning is a versatile method used for the preparation of PNCs through the insertion of nanomaterials such as metal nanoparticles, metal-oxide nanoparticles, carbon-based nanomaterials, and clay nanolayers into the polymer matrix [39,40]. For instance, electrospinning has been used for producing electrospun nanofiber mats of cellulose and organically modified montomontrille, which exhibited metal adsorption and removal of Cr^6+^ ions from aqueous solutions [41]. Electrospinning has also been used for preparing Ct nanocomposite fibers with multi-walled carbon nanotubes [42]. Similarly, PHBV nanocomposite fibers with multi-walled carbon nanotubes were produced using electrospinning. The rotating disc collector provides an extra drawing force for the stretching and aligning of the nanomaterial with the electrospun fibers [43,44,45]. Furthermore, cellulose/multi-walled carbon nanotubes nanocomposite was prepared through electrospinning using 14.25 kV of DC voltage and a horizontally positioned metal needle at the temperature of 27 °C and the relative humidity of 34% [44].

### 2.5. Other Methods

**Figure 5:**
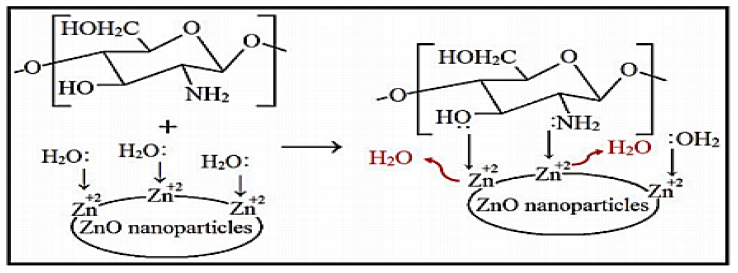
Complexation reaction between chitosan functional groups and Zn^+2^ ions of ZnO nanoparticles (taken from [46]).

One of the other methods reported for the preparation of PNCs is microwave heating. In one study, Ct/ZnO nanocomposite was prepared using microwave heating via a complexation reaction between the surface zinc cations of ZnO nanoparticles and the Ct functional groups. The optimum conditions for the preparation process were 800 watts of power and process duration of 10 min. The hydroxyl and amine groups of Ct served as a Lewis base, which formed coordination bonds with the surface zinc ions. As illustrated in Figure 5, the complexation reaction between the Ct functional groups and the surface of the ZnO nanoparticles was achieved via a ligand substitution reaction in which the Ct functional groups substituted water molecules (or the products of protolysis) coordinated to the surface Zn^+2^ ions [46]. In a similar report, a nanocomposite comprising Ct and magnetic nanoparticles was prepared by performing the functionalization of nanoparticles with carboxylic groups, which allowed the covalent bonding of the nanoparticles with the Ct amine groups [47]. In another report, Ct/TiO_2_ nanocomposite was prepared via the outer-sphere complexation reaction between the positive charges of the Ct chains and the negative charges present on the surface of TiO_2_ nanoparticles [31]. A related study reported the preparation of starch/multi-walled carbon nanotubes via covalent bond formation between the hydroxyl groups of starch and the groups present on the surface of the nanomaterial [48].

When comparing the different methods available for the preparations of PNCs, the physical preparation methods such as solution mixing and melt blending could prove to be advantageous as these are applicable to large-scale production as well. Moreover, these methods require a short duration for attaining the products and involve the use of a limited number of chemicals compared to the in situ synthesis technique. On the other hand, the in situ synthesis method is preferred when uniform and well-defined materials containing strong bonds are desired. However, the in situ synthesis method involves the use of several chemicals and glasses and requires high energy and a long duration for producing even small amounts of materials. On the contrary, the solution mixing method does not require high energy or a long duration for obtaining the products when used in large-scale production. In comparison, the melt blending method requires higher energy, shorter duration, and compounding devices without involving the use of solvents, thereby rendering it further advantageous compared to all other methods in terms of the limited number of chemicals used. In addition, melt blending provides rapid and continuous production of PNCs at an industrial scale. Electrospinning is much more complex compared to any of the other methods as it requires complex equipment and set-up. However, it produces nanocomposite fibers in the nanoscale dimensions with favorable properties. 

## 3. Smart Polymer Nanocomposites

**Figure 6:**
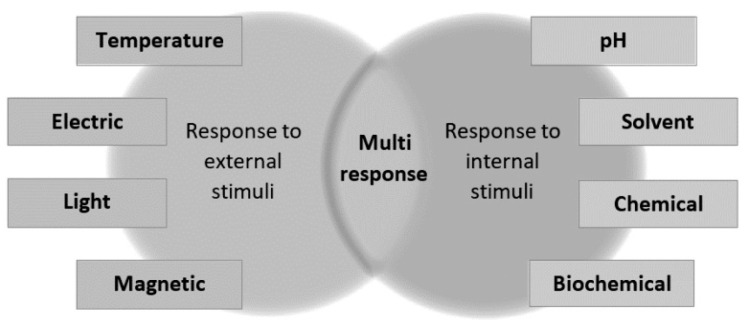
Classification of the responsive nanocomposite (taken from [9]).

Smart polymer nanocomposites or stimuli-responsive polymer nanocomposites are those that possess a minimum of one chemical and physical property that is controlled by or follows a stimulus-response. These stimuli-responsive properties may be categorized based on their nature as external (physical stimuli) and internal (chemical stimuli). Figure 6 presents the classification of responsive nanocomposites. The chemical stimuli are associated with the pH, biological recognition, solvent type, and chemical recognition, while physical stimuli are related to the magnetic field strength, temperature, electric current, and light [2,9]. These stimuli are discussed in the sections ahead.

### 3.1. Thermo-Responsive Nanocomposites

**Figure 7:**
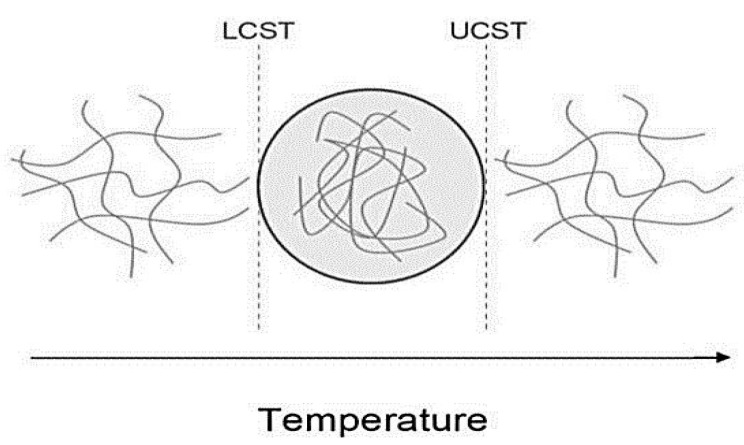
Reversible phase transition of thermo-responsive polymers (taken from [9]).

Thermo-responsive nanocomposites are commonly used as intelligent materials as these are based on the application of temperature as a facile stimulus. A thermo-responsive polymer exhibits a variation in the hydrogen bonds within the matrix. Hydrogel is a kind of thermo-responsive polymer that exhibits a switch from the hydrolyzed phase to the precipitated phase, which is accompanied by a considerable shift in volume in response to a difference in the temperature [49]. The reversible phase transition of thermo-responsive polymers is illustrated in Figure 7. The switch phase of the polymer occurs when the temperature lowers, and the transition temperature is referred to as the upper critical solution temperature (UCST) (including the copolymers of poly(acrylamide) and poly(acrylic acid)) [50]. On the other hand, the transition phase occurs as the temperature becomes high, and this temperature threshold is referred to as the lower critical solution temperature (LCST) [including poly(N,N-dimethyl acrylamide) and poly(N isopropyl acrylamide)] [51,52]. Nanofillers may also be added to improve the mechanical behavior and the thermo-responsiveness of the polymer matrix, properties that would extend the application of these polymers to be used as a tissue substitute. Various fabricated PNCs have been used as bone substitutes. For instance, Nistor et al. reported the fabrication of collagen/PNIPAAm hydroxyapatite nanocomposite to be used as an artificial extracellular matrix as this nanocomposite exhibited remarkable properties for bone tissue replacement above 33 °C [52]. Oguz et al. prepared methylcellulose-gelatin hydrogels with several calcium phosphate fillers. These hydrogels could be used as injectable nanocomposites that would shift to a solid phase inside the human bone tissue. Another interesting application is the utilization of the dual behavior of thermo-responsive polymers, one of which is the antibacterial effect exhibited by certain metal nanoparticles components of the nanocomposites [53]. Bacteria are capable of rapid growth, which renders their control difficult within the short duration available during the application of drugs. In this context, Arafa et al. fabricated the pluronic 127/gold nanocomposites release system for in vivo application, i.e., to be applied on skin burns [54].

### 3.2. Light-Responsive Nanocomposites

Light is simply produced or applied directly from solar energy. Light is not affected by electromagnetic fields as it is a source of remote stimulation. In addition, nanofillers, particularly metal-oxide nanoparticles, exhibit an important property of localized surface plasmon resonances (LSPR) through the interaction between the surface of particles and the light source [55,56,57]. The shape, nature, and size of the nanomaterial determine LSPR. This property leads to the production of intense electromagnetic fields upon the incidence of light and the occurrence of rapid heat transfer from the nanoparticles to the surrounding environment. It is the diversion to heat release which could produce the response of the polymer matrix, and this type of material is, at certain times, referred to as plasmonic polymer composite. Light-responsive nanocomposites have been studied extensively for application in the field of medicine, including cancer therapy and drug delivery. Among these applications, near-infrared irradiation (NIR) could be a remarkable candidate as it has deep permeation and negligible absorbance in the human tissue, properties that are not possessed by the other electromagnetic irradiation techniques such as those involving UV irradiation. Li et al. performed partial decomposition that was activated through the interaction of NIR with reduced graphene, thereby developing polyethyleneimine functionalized with thiocarbamate as a hydrogen sulfide (H_2_S) release platform. The exogenous H_2_S was then investigated for application in cancer therapy and tumor generation [58]. Raza et al. performed drug delivery using nanocomposites responsive to NIR for cancer therapy and investigating tissue regeneration [59]. Yang et al. studied the cell-growth promoting and bacteria-inhibiting capabilities of nanocomposites in skin lesions. Moreover, a near-infrared photo-responsive dressing nanocomposite based on a dodecyl-modified and Schiff’s base-linked Ct hydrogel, tungsten disulfide nanosheets (photothermal agent), and ciprofloxacin (antimicrobial drug) has been fabricated. This nanocomposite exhibited several advantages, including the ability to mold rapidly, self-adaptability, and injectability. The nanocomposite also exhibited good tissue adherence and excellent biocompatibility [60]. Yue et al. used graphene quantum dots as active NIR nanofillers in the dextran modified with pendant PNIPAAm chains to fabricate a nanocomposite. The nanocomposite comprised buprenorphine and thermo-responsive chains, which played a significant role in drug release [61].

### 3.3. Responsive Nanocomposites Based on Electric Current

The application of electric current in shape memory composites (SMC) has demonstrated promising results. When electrical current is applied, depending on the local heat release, a shift is produced in the shape of materials [62]. The most interesting choice here would be to use nanofillers as the active component, particularly carbon-based nanomaterials such as carbon nanotubes (CNTs), graphene oxide (GO), or graphene. The use of nanofillers would confer the property of responding to the electrical current stimulus due to the good thermal and mechanical behaviors of these nanofillers and their ability to significantly enhance the electronic behavior of the nanocomposite material. However, this property relies on the randomized distribution of the nanofillers within the matrix [63]. Yang et al. performed the in situ polymerization of graphene oxide nanoplatelets to fabricate a nanocomposite comprising 2-acrylamide-2-methyl propane sulfonic acid, acrylamide, and reduced graphene oxide. In comparison to pure polymer, these nanocomposites exhibited enhanced mechanical and electrical response behaviors, which facilitated their application as soft robots [64].

### 3.4. Magnetic Responsive Nanocomposites 

Magnetic nanoparticles (MNPs) exhibit magnetic induction heating, which facilitates their application in biomedical fields, including targeted drug delivery and elastomer fabrication [65,66,67]. The performance of magnetic induction heating relies mainly on the properties of the MNPs and the applied magnetic field conditions. The size of the nanoparticles affects their magnetic domains. While small sizes are constituted of a single domain, the larger sizes are constituted of multiple domains that result in the minimization of the magnetostatic energy. Moreover, in the field of cancer therapy, the conversion of magnetic energy into heat energy within the MNPs has demonstrated outstanding potential [68,69]. The facile separation and controlled placement of functionalized MNPs through the external application of magnetic field enabled the use of these MNPs in various bio-separation and catalytic processes. 

MNPs have been applied widely in biomedical fields owing to their superparamagnetic behavior and high biocompatibility. MNPs are suitable candidates for drug delivery agents and heat mediators in hyperthermia cancer treatment. MNPs could also serve as diagnostic and therapeutic agents in magnetic resonance imaging (MRI) [70,71]. The MRI technique relies on the counterbalance between the exceedingly large number of protons in the biological tissue and the small magnetic moment of a proton which undergoes a shift under the effect of a magnetic field. MNPs are influenced by static or alternating magnetic fields (AMF) with a relatively high penetration depth and non-contact stimulation source. The interaction between the magnetic gradient produced by the magnetic field and the magnetic moments in the material results in the development of magnetic behavior. The energy of the AMF could be converted into heat using MNPs via two types of relaxation processes, namely Neel relaxation and Brownian relaxation. Neel relaxation occurs due to the re-orientation of the magnetization that results from the re-orientation occurring inside the magnetic core against the energy barrier. Brownian relaxation occurs due to the rotational diffusion of the whole particle within the carrier liquid [72,73]. In the presence of MNPs, the magnetite poly(dimethylsiloxane) (PDMS) nanocomposites were fabricated using magnetic induction heating. Under the effect of an AC magnetic field, heat was generated by the MNPs that were utilized for accelerating the polymerization process and curing the PDMS. The magnetite PDMS composites with enhanced thermal stability compared to conventional PDMS were produced without the use of a catalyst within a short duration, and these were termed thermally stable elastomers [73]. The unmodified iron oxide nanoparticles exhibit a high surface/volume ratio, which causes the magnetic dipole/dipole attraction, thereby leading to the formation of aggregated particles. The aggregation unstabilizes the colloidal solution, leading to the loss of the size-dependent properties of the nanoparticles (such as their superparamagnetic behavior). Stabilizers comprising polymers and surfactants may be utilized as a coating on the material surface for higher stability through electrostatic repulsions and steric effects. Various polymer-and organic material-shelled iron oxide nanoparticles have been fabricated using this approach [74]. 

The magnetic response PNCs could exhibit changes in their movement or shape. These PNCs could be utilized as magnetically separable materials for purification systems and localized drug delivery. Moreover, at the nanoscale, hyperthermia might occur in the magnetic material. This may be influenced by the AMF for releasing heat through the oscillation of the nanomagnetic filler associated with the Brownian and Néel relaxation processes. This behavior could be applied to shape memory composites, artificial muscles, killing cancer cells in tumors, and drug release. Soto et al. fabricated polyurethane/Fe_3_O_4_ nanocomposites and also investigated the influence of the Fe_3_O_4_ content on shape recovery and heat release when these nanocomposites were exposed to AMF. It was revealed that the time taken to achieve shape recovery was directly correlated with the Fe_3_O_4_ content [65]. In another work, nanocomposites comprising Kappa carrageenan-g-poly(acrylic acid)/SPION were observed to be effective as in vitro antibacterial agents with the drug efficiency of 105 ± 8 µg/mg exhibited by the drug carrier [75]. SPION/polyvinyl alcohol/PMMA nanocomposites have been used for the delivery of ciprofloxacin. An increased release was achieved by applying a magnetic field, with high PMMA content, and with low PVA content [76]. Nanocomposites comprising PEG/phospholipid-coated iron oxide nanoparticles modified with peptide and a fluorescent dye were used for the confocal imaging of kidney-derived cells and primary human dermal fibroblast cells. These nanocomposites exhibited great potential in tissue imaging [67]. A polyethyleneimine/folic acid-targeted Fe_3_O_4_ nanocomposite was investigated for application in the in vivo MRI of tumors. This nanocomposite exhibited a high T2 relaxivity of 99.64 mM^−1^ s^−1^ when used as the nanoprobe for conducting the MRI of cancer cells [66]. 

**Figure 8:**
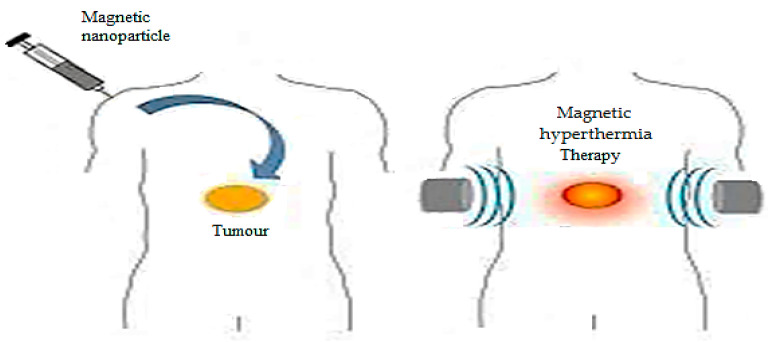
Artificially induced hyperthermia (taken from [77]).

Hyperthermia may occur on the MNPs, and it could be affected by the AMF for releasing heat through the oscillation of the MNPs associated with the Brownian and Néel relaxation processes. Figure 8 depicts a simple representation of artificially induced hyperthermia. Hyperthermia occurs when the temperature of the living tissue elevates beyond the physiological normal values. Hyperthermia is used in cancer therapy for damaging and killing cancer cells. It is also used for inducing local drug release from thermo-sensitive vehicles. Artificially induced hyperthermia involves locally elevating the temperatures of the influenced cells in the body up to 42 °C. It is used for specifically targeting and destroying the cancer cells without influencing the surrounding healthy cells [77,78]. A study investigated the development of injectable 20 nm dextran coated with iron-oxide nanoparticles and covalently bound antitumor chimeric L6 monoclonal antibody [79,80]. Using the co-precipitation method, modified magnetite nanoparticles exhibiting antibacterial and self-healing properties were prepared. Three sets of these nanoparticles were evaluated for their antibacterial properties and magnetic heating specific absorption rates. The determined concentration of these nanoparticles for 10% growth inhibition (EC10) of *S. aureus* and *E. coli* was 150 mg/L [81].

## 4. Applications of Polymer Nanocomposites

### 4.1. Water Treatment 

Recently, PNCs have been receiving great attention in the field of wastewater treatment and remediation. Freshwater sources have been demonstrating a continuous decrease in both quantity and quality, mainly due to anthropogenic activity. Water is essential for humans and plants and is also a primary resource in various industrial processes. Owing to rapid industrialization, the discharge of wastewater has increased greatly, which has led to diverse pollutants being released into the environment and causing adverse effects on the environment as well as human health. In this regard, PNCs could serve as an efficient and cost-effective material for wastewater treatment. PNCs appear to be promising in resolving the inherent challenges of ordinary particles when used in water treatment [82]. Nanocomposites comprising polymers integrated with nanomaterials exhibit improved properties, such as enhanced resistance to fouling, thermal stability, membrane permeability, good mechanical behavior, higher photocatalytic activity, and higher adsorption [83]. The extent of these properties depends on the type of nanoparticle incorporated, its shape and size, its interaction with the polymer, and its concentration. Moreover, the method used for the modification of PNCs determines their reusability, remediation capacity, and selectivity. PNCs could be used in the field of water treatment to achieve various purposes, including the removal of dyes, metal ions, and microorganisms from water [84]. The roles of PNCs in dye removal, metal removal, and water disinfection are discussed in the sections ahead.

#### 4.1.1. Dye Removal

**Figure 9:**
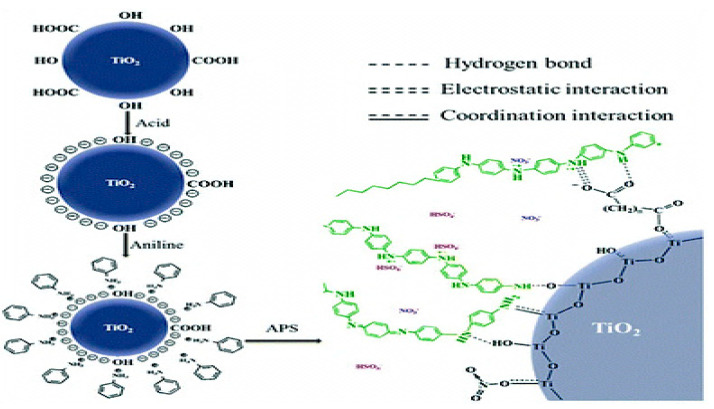
Schematic representation of the adsorption mechanisms by polyaniline/TiO_2_ nanocomposite (taken from [89]).

Dyes are used widely in several industries, such as textiles, paper making, ink, coating, and cosmetics, to induce color into the products. However, severe environmental problems are caused due to the wastewater discharged from these industries, which is polluted with dyes that are carcinogenic, toxic, and hazardous. Therefore, it is essential to remove these dyes for rendering the wastewater suitable for industrial reuse and also to prevent environmental impact. In this regard, several studies have used PNCs as efficient materials for the removal of dyes from polluted water [85,86,87,88,89,90,91,92,93,94,95,96,97,98,99,100,101,102,103,104,105,106]. This ability of PNCs is conferred by the synergistic effect of the polymer and the nanomaterial [86]. Table 1 lists a few examples of PNCs that have been utilized for dye removal. PNCs used as adsorbents for dyes exhibit a large surface area, numerous active sites, and chemical and thermal stability. For instance, Zaman et al. fabricated the cellulose/graphene oxide nanocomposite for the enhanced removal of methylene blue dye through adsorption. Approximately 98% of the dye was removed within 135 min, with the nanocomposite achieving an adsorption capacity of 334.19 mg/g [87]. In another work, the methylene blue dye was removed using cellulose/clay nanocomposites and the maximum removal efficiency achieved was 98% [88]. When the polyaniline/TiO_2_ nanocomposite was used for the removal of methylene blue dye removal in a different study, the maximum adsorption capacity achieved was 458.10 mg/gL. The adsorption was achieved through membrane diffusion, chemical adsorption, and intraparticle diffusion, and hydrogen bonding, coordination interaction, and electrostatic interaction were revealed as the adsorption mechanisms, as illustrated in Figure 9 [89].

In addition, certain PNCs comprising polymer and nanomaterial photocatalysts such as the nanoparticles of ZnO, TiO_2_, and CuO, have been used for dye removal. The presence of these photocatalysts degrades the dyes as these photocatalysts produce hydroxyl free radicals that are capable of degrading organic materials. Therefore, when using PNCs for dye removal, the combination of the adsorption process via the active sites of PNCs and the photocatalytic degradation of the dye due to the action of the photo-catalyst appears to be the most suitable approach [90]. For instance, a study evaluated the efficiency of Ct and Ct/ZnO in methylene blue dye removal and reported the values of 81% and 96.7% of the methylene blue dye removed by Ct and Ct/ZnO, respectively. The enhanced dye removal by the nanocomposites was attributed to the synergistic effect of the adsorption process accomplished by the nanocomposites and the photocatalytic activity of the ZnO nanoparticles [46]. In another study, Kumar et al. fabricated a Ct/CuO film to be used as a simple, portable, recoverable, reusable, and efficient photocatalyst. Using these nanocomposites, up to 99% of Rhodamine B dye was removed within 60 min of irradiation because of the slow recombination rate of the electron–hole pair of the CuO nanoparticles in the chitosan matrix [91]. Shaikh et al. fabricated the PLA/TiO_2_ nanocomposites and investigated their efficiency in degrading malachite green and methyl orange under UV light and solar light. In comparison to the UV light conditions, the degradation was faster under the sunlight. In sunlight, a 10^–4^ M solution of each of the two dyes was completely decolorized within 8 min and 20 min, respectively [92]. Karagoza et al. prepared polycaprolactone/Ag/TiO_2_ nanocomposites to be used as photocatalysts for the degradation of organic pollutants. The fabricated nanocomposites were able to completely degrade methylene blue and ibuprofen under UV irradiation within approximately 180 min [93]. Meenakshi et al. reported that Ct/TiO_2_ nanocomposites, via photodegradation, could remove Reactive Red 2, methylene blue, and Rhodamine B [94]. 

PNCs that contain magnetic nanoparticles may be used as reusable magnetic adsorbents for dyes by exploiting the magnetic properties conferred by their component nanoparticles. The PNC magnetic adsorbents exhibit a large surface area, porous structure, and small particle size, and their excellent magnetic properties allow convenient recovery via magnetic separation after the adsorption or regeneration. This enables overcoming the limitations of separation difficulty encountered when using PNC adsorbents while increasing the reusability of the PNCs in dye removal. For instance, the magnetic Ct nanocomposites prepared using the chemical approach were applied as a reusable adsorbent for dyes, and an adsorption capacity of 20.5 mg/g, based on the pseudo-second-order model, was achieved [95]. Esvandi et al. prepared a magnetic nanocomposite using starch, clay, and MnFe_2_O_4_ for the uptake of sunset yellow and Nile blue dyes from water [96]. Guan et al. developed a nanocomposite containing cellulose nanocrystals and zinc oxide for the removal of malachite green and methylene blue dyes. This nanocomposite exhibited rapid removal and high dye removal efficiency, with high dye-removal ratios for malachite green (99.02%) and methylene blue (93.55%) [97].

**Table 1:** Performance of some polymer nanocomposites in dye removal.

Polymer Nanocomposite	Dye	Results	Ref.
Chitosan/CuO nanocomposites beads	Congo red (CR) Eriochrome black T (EBT)	A total of 97% of dyes were removed within 2 h. Maximum adsorption capacity of CR and EBT were 119.70 and 235.70 mgg^−1^	[98]
Molecularly imprinted Chitosan/TiO_2_ nanocomposite	Rose Bengal (RB)	The adsorption capacity for RB was 79.365 mg/g and enthalpy was 62.279 kJ mol^−1^	[99]
Chitosan/ZnO nanocomposite	Methylene blue (MB)	96.7% of MB dye was removed	[46]
ZnO/Cellulose nanocrystal nanocomposite	Methylene blue (MB) Malachite green (MG)	93.55% and 99.02% of MB and MG were removed within 5 min. The absorption capacity was 46.77 and 49.51 mg/g for MB and MG	[97]
ZnO/Poly(methyl methacrylate) nanocomposite membrane	Methylene blue (MB)	About 100% of MB was removed within 80 min	[100]
Poly(methyl methacrylate)/Multiwall carbon nanotube nanocomposite	Methyl green (MG)	The Langmuir adsorption capacity for MG was 6.85 mmol/g at 25 °C	[101]
Polyacrylic acid/Fe_3_O_4_/Carboxylated cellulose nanocrystals nanocomposite	MB	The maximum adsorption capacity for MB was 332 mg g^−1^	[102]
Fe_3_O_4_/Starch/Poly (acrylic acid) nanocomposite hydrogel	Methylene violet (MV) Congo red (CR)	A maximum of 93.83% and 99.32% CR and MV dyes with maximum adsorption of 96.7% and 97.5%	[103]
Polylactic acid/Graphene oxide/Chitosan nanocomposite	Crystal violet (CV)	97.8 ± 0.5% of CV was removed	[104]
Polypyrrole/Zeolite nanocomposite	Reactive blue (RB) Reactive red (RR)	A total of 86.2% of RB and 88.3% of RR were adsorbed from synthetic solution	[105]

Dye removal using PNCs may be improved by controlling several factors, such as the types and properties of the polymer and the nanomaterial. Moreover, the methods used for PNC preparation and the design of the fabricated PNC play essential roles in determining the performance of the fabricated PNC in dye removal. The mechanism of dye removal is also an important factor. For instance, selecting a PNC capable of removing dyes through a combination of the adsorption approach and the photocatalytic degradation approach would result in improved performance compared to the PNCs following either of the individual mechanisms. The PNCs possessing remarkable magnetic properties would be further advantageous as these may be recovered conveniently and then reused several times. In addition, the experimental conditions, including dye concentration, PNC concentration, pH, temperature, and contact duration, could affect the dye removal process. Therefore, the integration of all these factors would allow the fabrication of a PNC that would exhibit outstanding performance in dye removal [90]. Generally, it is the laboratories where the PNCs have been applied the most widely for dye removal. However, the scaling up and production in huge amounts for use at the industrial scale remains to be achieved so far and warrants further research and improvement on the production cost and practical application possibility. Several other issues are also required to be dealt with, including the following: (i) the toxicity of the PNCs has to be considered to prevent secondary pollution, (ii) greater efforts are required for the selective removal of a specific dye using the PNC in the presence of other dyes, (iii) PNCs with the ability to be regenerated several times and be used for longer durations should be investigated. In addition, PNCs that are environmentally friendly and highly stable, and also exhibit potential for large-scale production should be produced. The preparation of PNCs at a low cost is an important factor when considering them for large-scale applications. Moreover, efficient methods of separation of the PNCs from solutions after use should be developed to prevent the PNCs from acting as pollutants [106].

#### 4.1.2. Metal Ion Removal

**Figure 10:**
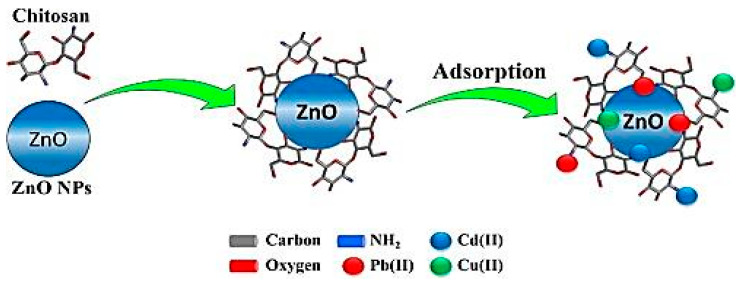
Schematic representation of metal ion adsorption by nanocomposite (Taken from [114]).

Metal pollution has been increasing rapidly throughout the world due to rapid advancements in urbanization and industrialization, which has led to serious environmental concerns. Metal ion removal has, therefore, become an essential requirement for the protection of the environment and human health. In this regard, PNCs could be useful in metal ion removal. Table 2 lists the performance of a few PNCs in metal ion removal. PNCs may remove metal ions through the adsorption process, which is a facile and efficient mechanism for the removal of heavy metal ions, such as Cu (II), Cd (II), Pb (II), Co (II), Cr (VI), and Ni (II), from solutions. The adsorption of metal ions on a PNC may be affected by several factors, including the concentration of the adsorbent, pH of the solution, the concentration of metal ions in the solution, contact duration, and temperature conditions. In comparison to individual polymer adsorbents, PNCs exhibit a greater number of surface groups available for interaction, a larger number of active sites, better stability, mechanic feasibility, and higher adsorption capacity [107]. Several studies have reported the use of PNCs in metal ion removal. For instance, Cr (VI) was removed using polyaniline β-FeOOH prepared through the blending process (mechanical force) [108]. A related study demonstrated that the magnetite acrylamide amino-amidoxime nanocomposites prepared using the in situ method exhibited excellent sorption properties and could, therefore, be utilized for treating U(VI) present in aqueous solution [109]. In another work, magnetite/poly (1-naphthylamine) nanocomposite was prepared using the in situ method and then used for As (III) removal [110]. Another nanocomposite composed of poly(N-vinylcarbazole) and graphene oxide was fabricated in a study and then used for adsorbing heavy metals from aqueous solutions. The Pb^2+^ adsorption capacity of this nanocomposite could be increased by increasing the graphene oxide content of the nanocomposite due to the resulting increase in the concentration of oxygen-containing groups in the nanocomposite. The highest Pb^2+^ adsorption capacity achieved using this nanocomposite was 887.98 mg g^−1^, and the adsorption fitted well with the Langmuir model [111]. Another study reported the fabrication of polyethersulfone/Fe-NiO nanocomposites through solution blending, followed by the application of these nanocomposites for salt removal [112]. A novel nanocomposite comprising poly(methyl methacrylate)-grafted alginate/Fe_3_O_4_ was synthesized through oxidative-free radical-graft copolymerization reaction and then used for the adsorption of Pb^2+^ and Cu^2+^ ions from aqueous media. The pH value of 5 was determined as the optimum condition for the adsorption process in the afore-stated study, and the maximum adsorption capacity achieved for Cu^2+^ and Pb^2+^ ions was 35.71 mg g^−1^ and 62.5 mg g^−1^, respectively [113]. A. A. Saad reported using ZnO/chitosan/organically nanocomposite for the removal of Cu(II), Cd(II), and Pb(II) ions from polluted water [114]. Figure 10 depicts the schematic representation of the adsorption of metal ions using a nanocomposite.

Owing to advantageous properties such as high adsorption capacity, great recycling performance, high mechanical strength, and convenient separation from the solution, PNCs have been applied widely for the removal of metal ions from wastewater. The polymer present in a PNC serves as a support for the nanoparticles and also as a chelating material, while the incorporated nanoparticles exhibit effective chelating sites, reducibility, and exceptional magnetic properties. PNCs demonstrate rapid adsorption kinetics, remarkable regeneration capability, and strong chelating abilities for metal ions. Unfortunately, scaling up becomes an issue for these PNCs, and their preparation methods have not been adapted for scaling up from laboratory to industrial level so far. Moreover, the research on production cost and practical application possibilities is scarce. Several other issues also require investigation, such as (i) evaluation of the adsorbent capacity for each metal ion to compare different kinds of adsorbents, (ii) development of reliable approaches for adsorbent regeneration, and (iii) exploring PNCs that exhibit long-term performance and better regeneration capability. In addition, further clarification of the adsorption mechanism using advanced analytical and characterization techniques is required to gain deeper insights into the molecular mechanisms underlying the metal ion removal process and the interaction process [115,116].

**Table 2:** Performance of some polymer nanocomposites in metal ion removal.

Polymer Nanocomposite	Metal Ion	Results	Ref.
Polyaniline/Reduced graphene oxide nanocomposite	Hg(II)	The adsorption capacity was 1000.00 mg/g	[117]
Fe_3_O_4_/starch/Poly(acrylic acid) nanocomposite hydrogel	Cu(II) Pb (II)	95.4% of Cu(II) and 88.4% of Pb(II) were removed at pH of 6.0 and 5.5	[103]
Graphene oxide/Chitosan/ Ferrite nanocomposite	Cr (VI)	The adsorption capacity for Cr(VI) was 270.27 mg g^−1^ at pH of 2.0.	[118]
Magnetic chitosan/Functionalized 3D graphene nanocomposite	Pb (II)	The efficiency of Pb(II) removal is 100% at pH of 8.5 within 18 min	[119]
Bacterial cellulose/Amorphous TiO_2_ nanocomposite	Pb(II)	A total of 90% of Pb(II) was removed in 120 min at pH 7	[120]
Cellulose/TiO_2_ nanocomposite	Zn(II) Cd(II) Pb(II)	Maximum adsorption capacity for Zn(II), Cd(II) and Pb(II) was 102.04, 102.05 and 120.48 mg/g	[121]
Polyacrylamide/Sodium Montmorillonite nanocomposite	Ni (II) Co (II)	A total of 99.3% of Ni(II) and 98.7% of Co (II) was removed at pH 6.	[122]
Polyacrylamide/Bentonite hydrogel nanocomposite	Pb (II) Cd (II)	More than 95% of Pb (II) and Cd (II) were removed within first 20 min. Maximum adsorption capacity for Pb (II) and Cd (II) was 138.33 and 200.41 mg/g.	[123]
Modified mesoporous silica/Poly(methyl methacrylate) nanocomposites	Cu (II)	Maximum adsorption capacity for Cu (II) was 41.5 mg/g at pH 4 and 140 min	[124]
Xanthan gum grafted Polyaniline/ZnO nanocomposite	Cr(VI)	Maximum adsorption capacity was 346.18 mg g^–1^ for Cr(VI)	[125]

#### 4.1.3. Water Disinfection

The pathogenic microorganisms present in drinking water affect human health greatly. Therefore, disinfection of water is necessary, either through the deactivation or by complete removal or killing of the pathogenic microorganisms. In this regard, PNCs exhibiting antimicrobial activity could be utilized for inhibiting the growth of microorganisms present in drinking water [83]. The good antimicrobial activity of PNCs is a consequence of the synergistic effect of its component polymer and nanomaterial. Several studies have reported the use of PNCs as antimicrobial agents for water disinfection. For instance, Chen and Peng prepared a cellulose/silver nanocomposite that exhibited improved antimicrobial activity and high water permeability, which rendered it suitable for application as an antimicrobial agent in the field of water treatment [126]. Sarkandi et al. fabricated a nanocomposite hydrogel comprising cellulose and silver nanoparticles, which exhibited excellent antibacterial activities and 100% reduction in bacterial percentage, while inhibition zones of 2.8 cm and 2.6 cm against *E. coli* and *S. aureus*, respectively, were observed [127]. Munnawar et al. prepared the chitosan/zinc oxide nanocomposite and incorporated it into a polyethersulfone matrix to develop antifouling polyethersulfone membranes, which exhibited outstanding water permeability and prevented microbial fouling [24]. In another study, a polycaprolactone nanocomposite membrane with ZnO nanoparticles was fabricated, which demonstrated enhanced antimicrobial activity against *S. aureus* and *E. coli* [128]. Al-Naamani et al. used a chitosan/ZnO nanocomposite coating to prevent marine biofouling. The nanocomposite used demonstrated antibacterial activity against the marine bacterium *Pseudo alteromonasnigrifaciens* and anti-diatom activity against *Navicula* sp. [129]. In another study, a nanocomposite of polypyrrole/carbon nanotubes/silver was prepared through the in situ oxidative polymerization of pyrrole with AgNO_3_ containing single-wall carbon nanotubes. The nanocomposite was then used for the inhibition of bacteria in water, and the *E. coli* removal percentage achieved was 87.5–95% [130].

Although the excellent antimicrobial behavior demonstrated by PNCs when used in water disinfection has rendered PNCs effective against different kinds of microorganisms, further investigation is nonetheless warranted in this field of research. For instance, efforts for large-scale PNC production at lower costs have to be increased and the practical application potential of these PNCs in actual-world scenarios has to be ensured. Several other issues have to be addressed as well, such as (i) the preparation of PNCs with excellent antimicrobial activity against a higher number of microorganisms and (ii) the investigation of the antimicrobial activity of PNCs using further advanced techniques to attain deeper insights into the mechanism of their antimicrobial activity. 

### 4.2. Sensor Devices

Sensors have a wide range of applications, including the detection of chemicals and toxic gases for safety purposes, medical diagnosis, and defense applications. In order to be effective, a sensor must have small dimensions, multiple functions, low cost, reliability, rapid response, higher sensitivity, and selectivity. Among these qualities of sensors, rapid response and high sensitivity are achieved with a large specific surface area. In this regard, polymer nanocomposites could be considered promising candidates for fabricating sensors [131]. The use of PNCs in the fabrication of sensors has become common these days. This is because polymeric materials are inexpensive and convenient to fabricate and also exhibit multi-functionality owing to their varied physicochemical and structural behaviors. Moreover, the modification of sensors, such as the addition of side chains and inorganic materials into the bulk matrix, is convenient when using PNCs. The resultant behaviors include enhanced conductive, electrolytic, dielectric, and optical properties, better ion selectivity, and improved molecular recognition capability [132]. The nanofillers used in the preparation of PNCs aimed at sensing applications must possess certain unique behaviors, such as electrochemical, optical, and magnetic properties, for enhancing the sensitivity and detection rate of the sensors.

**Figure 11:**
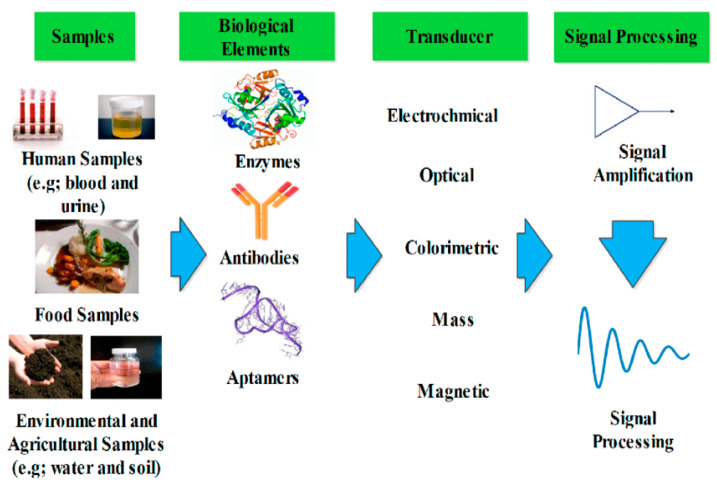
Schematic representation of biosensor (taken from [132]).

**Figure 12:**
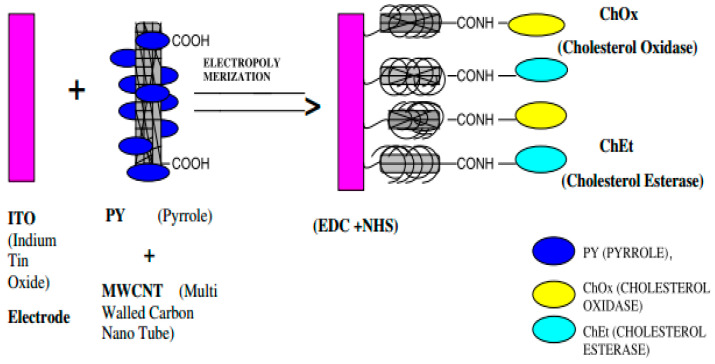
Fabrication of nanocomposite electrode (taken from [138]).

PNCs have been used widely in various kinds of sensors, including biosensors, gas sensors, and metal ions sensors. Table 3 summarizes the performance of a few PNCs in different sensors. A biosensor is capable of interacting with biological components and thereby detecting biomolecules, including cholesterol, glucose, and DNA. Figure 11 depicts the setup of a biosensor. As visible in the figure, a biosensor comprises a biologically active element that is immobilized on a convenient substrate, a transducer, and a signal processor. The biologically active element may be an enzyme, DNA, or a protein [133]. PNCs have been used as bio-sensing materials for the fabrication of biosensors. For instance, a sensor relying on poly (3,4-ethylenedioxylthiopene)/Au nanocomposite was used for measuring the concentration of 17β-estradiol using square wave voltammetry and cyclic voltammetry. In this biosensor, the transduced signal released was lower because of the interference of bound 17β-estradiol, while the current drop was proportional to the concentration of the contaminant. In addition, the probe exhibited outstanding selectivity as it could distinguish 17β-estradiol from the other structurally similar EDCs [134]. Narang et al. fabricated a NiO–chitosan/ZnO/zinc hexacyanoferrate film to be used as a triglyceride biosensor. When this biosensor was polarized at +0.4 V against Ag/AgCl, its optimum response was obtained within 4 s at 35 °C and pH 6.0. Moreover, a linear relationship existed between the response of the sensor and the concentration of triolein in the concentration range of 50–700 mg/dL, and a sensitivity of 0.05 A/mg/dL was achieved [135]. In another report, cellulose/organic montmorillonite nanocomposites were fabricated for use in bio-macromolecular quorum sensing inhibitors, which were capable of interfering with the quorum-sensing-regulated physiological process of bacteria. This would provide a sustainable and inexpensive approach for dealing with the challenges raised due to microbial infections in numerous products, such as biomedical materials or food packaging [136]. Manno et al. prepared starch/Ag nanocomposites using the green method and utilizing starch as the capping agent. The fabricated nanocomposites exhibited high sensitivity for hydrogen peroxide [137]. Singh et al. reported the fabrication of a nanocomposite electrode based on polypyrrole through the electrochemical deposition of carboxy functionalized multi-walled carbon nanotubes on an indium–tin–oxide (ITO) electrode via p-toluene sulfonic acid (PTS). Subsequently, cholesterol esterase and cholesterol oxidase were immobilized onto this nanocomposite electrode using N-ethyl-N-(3-dimethylaminopropyl) carbodiimide and N-hydroxy succinimide for the detection of cholesterol. Figure 12 presents the schematic representation of this fabricated nanocomposite electrode. The electrode exhibited a response time of 9 s, a linear concentration range of 4 × 10^−4^ to 6.5 × 10^−3^ M/L, and thermal stability up to 45 °C [138]. 

Gas sensors could be used for monitoring and controlling gas emissions from several emitters. A gas sensor comprises a sensing material capable of detecting combustible gases toxic gases, and vapors. PNCs have been utilized in gas sensors due to their properties of large surface area, high sensitivity, high selectivity, and good electrical conductivity. PNCs are able to detect various gases, including ammonia and chloroform vapors. For instance, PLA/multi-wall carbon nanotube-based conductive biopolymer nanocomposites were developed using the spray layer-by-layer technique. The chemo-resistive behavior of these nanocomposites was studied based on exposure to various organic vapors (methanol, chloroform, water, and toluene) exhibiting different physical properties. The results indicated that the largest response from these sensors was obtained for chloroform, demonstrating the ability of nanocomposites to serve as a vapor sensor [139]. Zhu et al. studied a sensor based on the nanocomposites composed of carbon nanotubes and cellulose, which was highly sensitive to humidity. The nanocomposites exhibited superior performance as a humidity sensor, achieving a maximum response value of 69.9% (ΔI/I0) at a relative humidity of 95%. The sensor also exhibited good long-term stability and bending resistance. In addition, the fabricated humidity sensor could be used for monitoring human breath [140]. Cheng reported a nanocomposite based on polycaprolactone/carbon black, which was used as a sensor for the detection of solvent vapors [141]. Pandey et al. prepared agar gum/silver nanocomposites, which when used as sensors, exhibited a response time of 2–3 s and an ammonia solution detection limit of 1 ppm at room temperature. These nanocomposites appear promising for use as optical sensors for ammonia detection [142]. Dai et al. prepared nanocomposites based on Ct/ZnO/single-walled carbon nanotubes to be used as chemo-resistive humidity sensors. The sensing was achieved owing to the Ct swelling behavior of the surrounding nanotubes, which led to a change in the hopping conduction path between the nanotubes [143]. Chakraborty reported the fabrication of PLA/exfoliated graphene nanocomposites to be used as a sensor for the detection of ethanol vapor [144].

The designing of the sensors for the detection of heavy metal ions is highly desired as heavy metal ions cause a great threat and hazards to the ecosystem. PNCs have been used in heavy metal ion sensors owing to their good conductivity and environmental stability. PNC-based sensors have been used for detecting several heavy metal ions, including cadmium ions (Cd^2+^), lead ions (Pb^2+^), and copper ions (Cu^2+^). For instance, Khachatryan et al. fabricated a sensor based on starch/ZnS quantum dots/L-cysteine nanocomposites for the detection of Pb^2+^ and Cu^2+^ ions [145]. Another sensor based on graphene oxide/carbon nanotubes/poly(O-toluidine) nanocomposite could selectively detect Pb^2+^ ions in aqueous solutions and also exhibited an antimicrobial behavior by inhibiting *E. coli* and *B. subtilis* [146]. Wang et al. used glassy carbon electrodes coated with polyaniline/multi-wall carbon nanotubes for the detection of Pb^2+^ ions in the buffer solution of acetate. The modified electrode exhibited enhanced activity compared to the original glassy carbon electrode [147]. Y. Shao et al. used a screen-printed carbon electrode modified gold nanoparticles/polyaniline/multi-wall carbon nanocomposite for fabricating a highly sensitive sensor for the detection of Cu^+2^ ions with the detection limit of 0.017 µg/L and a linear concentration range of 1–180 µg/L. The sensor also exhibited excellent stability, selectivity, repeatability, and reproducibility [148].

**Table 3:** Performance of some polymer nanocomposites in sensors.

Polymer Nanocomposite	Type of Sensor	Target	Results	Ref.
NiO– chitosan/ZnO/Zinc hexacyanoferrate nanocomposite film	Biosensor	Triolein	Optimum response: within 4 s linear concentration range: (50–700 mg/dL) Sensitivity: 0.05 A/mg/dL	[135]
GOx/MWCNTs-polyaniline nanocomposite.	Biosensor	Glucose	Electrical conductivity: 3.78 × 10^−1^ Scm^−1^ Response time: 5 s Linear concentration range: 0.5–22 mM	[149]
Polyaniline/MWCNTs/Au NPs nanocomposite modified glass carbon electrode	Biosensor	Glucose	Detection limit: 0.19 mM Sensitivity: 29.17 mA mM^−1^ cm^−2^ Concentration range: 0.0625–1.19 mM	[150]
Polypyrrole/MWCNTs/GOx nanocomposite modified glassy carbon electrode	Biosensor	Glucose	The linear range: up to 4 mM Sensitivity: 95 nAmM^−1^ Response time: 8 s	[151]
Polypyrrole/MWCNTs/Au NPs/ChOx	Biosensor	Cholesterol	Linear response: (2 × 10^−3^ to 8 × 10^−3^ M) Detection limit: 0.1 × 10^−3^ M Sensitivity: 10.12 mA mM^−1^ cm^−2^.	[152]
Polyaniline/ Functionalize MWCNT nanocomposite	Gas Sensor	Ammonia Vapor	High sensitivity (92% for100 ppm) Detection limit: (200 ppb) Response time: (9 s) Recovery time: (30 s)	[153]
Polypyrrole/Nitrogen-doped MWCNTs film fabricated on PI substrate	Gas Sensor	NO_2_ gas	The sensor possessed high response of 24.82% (Rg − Ra)/Ra × 100%) under 5 ppm of NO_2_. The sensor had outstanding selectivity, repeatability and stability	[154]
Ethylene diamine tetraacetic acid/Polyaniline/MWCNTs. with carbon electrode	Metal ion sensor	Pb^+2^	Detection limit: 22 pM	[155]
Polypyrrole/MWCNTs deposited on electrode	Metal ion sensor	Pb^+2^ ions	Detection limit: 2.9 × 10^−9^ mol/L (S/N = 3)	[156]
Polyaniline/MWCNTs -3-aminopropyltriethoxysilane casted on glassy carbon electrode	Metal ion sensor	Cd^+2^ ions	Detection limit: 0.015 µM Linear concentration range:(0.05–50 µM)	[157]
Modified glassy carbon electrode with polythiophene/COOH -MWCNTs/reduced graphene oxide	Metal ion sensor	Hg^+2^ ions	Linear range: (0.1 to 25 µM) Limit of detection: (0.009 µM) Recovery: between 110.7 and 96.79%	[158]

The use of PNCs as sensing materials in sensors is based on several advantages of PNCs in terms of stability, good electrical performance, and chemical properties. PNCs are able to detect various compounds, including biomolecules, gases, and metal ions. However, certain challenges regarding sensitivity, selectivity, and recovery times are encountered. Selectivity remains a major challenge when detecting specific species. The non-repeatability of certain PNCs limits their practical application potential in sensors as it would be economically unaffordable to use the sensor only one time. Factors such as the design of the PNCs, the type of polymer component of the PNC, the type of component nanofillers, and the ratio between the polymer and the nanofillers are important in determining the performance of a PNC as a sensing material. Among the different polymers, conducting polymers could be the most effective due to their remarkable performance as a sensing material. The carbon-based nanomaterials have also been used widely as nanofillers in the preparation of PNCs aimed for sensors as these nanomaterials possess excellent electrical properties. A clear understanding of the PNC properties, the type of interaction between the component polymer and nanomaterial, the effect of the size and shape of the PNC, the surface area of the PNC, its sensing mechanism, porosity, and other factors affecting the sensing capability would contribute immensely to the controlled synthesis of a PNC capable of fulfilling all the desired requirements. Therefore, further research is necessary to develop a PNC to be used as a sensing material that would exhibit improved performance at a lower cost compared to the currently available PNCs, which would, in turn, facilitate the expansion of the scope of application of PNCs in industrial fields [159]. 

### 4.3. Electromagnetic Shielding in Aerospace Applications

**Figure 13:**
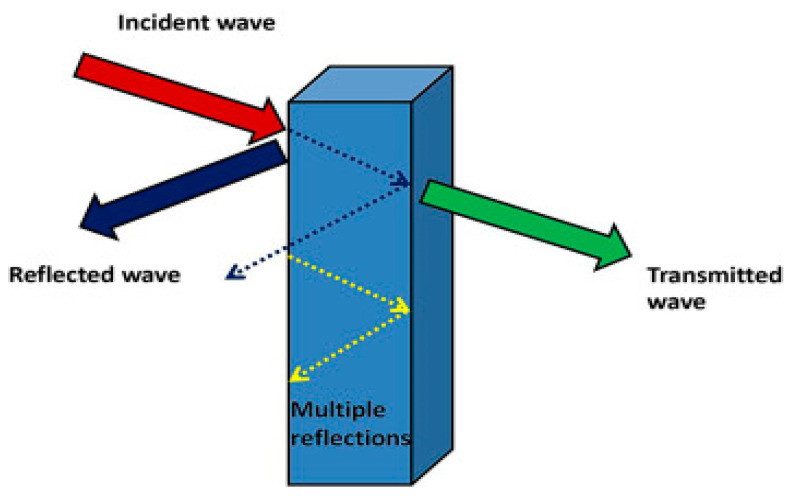
Schematic representation of EMI shielding (taken from [164]).

The advances in the technology and utilization of telecommunication devices and electronics have increased electromagnetic pollution. The resulting electromagnetic interference (EMI) could disrupt equipment, systems, and the electronic devices used in critical fields, such as military, aerospace, and medicine. Long-term exposure to electromagnetic waves could cause adverse effects on human health. Electronic systems and equipment generate waves that exist in the microwave range of the electromagnetic radiation spectrum. These radiations have to be shielded. In this context, electromagnetic shielding is defined as the practice of electromagnetic field reduction in a space using a blocking field with barriers composed of a magnetic or conductive material. Shielding is achieved within enclosures used for isolating the electrical devices from the “outside world” and isolating the wires from the environment through which the cables run [160]. Figure 13 presents the schematic representation of EMI shielding. The mechanisms that are exploited for shielding could be categorized into three main classes—reflection, absorption, and multiple reflections. Reflection is the primary mechanism exploited in EMI shielding. In order to shield radiation through reflection, the shield has to contain the mobile charge carriers—either electrons or holes—that would interact with the electromagnetic field in the radiation to be shielded. Absorption is the secondary mechanism of EMI shielding. In order to shield radiation mainly through absorption, the shield must have magnetic or/and electric dipoles that would interact with the electromagnetic field in the radiation. The electric dipoles could be provided by BaTiO or other materials with a high value of the dielectric constant, while the magnetic dipoles could be provided by FeO or other materials with a high degree of magnetic permeability. The third mechanism of EMI shielding is multiple reflections, in which reflections occur at various interfaces or surfaces in the shield. This mechanism of shielding requires a large interface or surface area in the shield. The loss owing to multiple reflections is negligible when the distance between the reflecting interfaces or surfaces is large compared to the skin depth. Shielding effectiveness (dB) of a material is defined as the sum of all the losses that have occurred due to reflection, absorption and multiple reflections [161,162,163].

The effectiveness of shielding relies on conductivity; higher conductivity leads to better shielding efficiency. The development of lightweight materials with high electromagnetic radiation shielding performance to enable the prevention of interference is highly desirable. In this regard, PNCs could serve several purposes, which include providing solutions for aerospace and environmental applications. Various PNCs that comprise conductive nanofillers, such as metal nanoparticles, magnetic nanoparticles, or carbon nanomaterials, could be utilized as EMI shielding materials. Table 4 lists the shielding performance of a few lightweight polymer nanocomposites. In a study, polyvinylpyrrolidone/Fe_3_O_4_ nanocomposite nanofiber (FCNF) was developed, and its capability as electromagnetic interference (EMI) shielding material was studied using the frequency range of 8.2–12.4 GHz. The EMI shielding efficiency of FCNF increased up to approximately 22 dB, demonstrating that reflection was the secondary shielding mechanism, while the major shielding mechanism was absorption [165]. In another study, electromagnetic shielding composites based on the acrylic resin matrix (AR) were fabricated by inserting up to 30% (wt.%) activated charcoal (AC) loading. It was observed that the 30% (wt.%) AC loading composite exhibited a higher relative permittivity value (∼79) compared to the AR (∼5). Moreover, the electrical conductivity, porous structure, and permittivity value of the composite contributed to the EMI shielding effectiveness value of −36 dB, revealing the ability of these composites to serve as an efficient coating for EMI shielding [166]. The porous polymer nanocomposites fabricated in a study through the ionic self-assembly of gold nanoparticles on the charged polymer skeleton composed of poly (pyridobisimidazole)-grafted-poly(dimethyl diallyl ammonium chloride) exhibited a shielding effectiveness value of over –64.9 dB in the frequency range of 250 MHz–1.5 GHz with a thickness of only 20 mm [167].

The EMI shielding fabrics based on polyaniline and its composites and fabricated using the in situ polymerization method exhibited the properties of polyaniline and its composites as well as those of the fabrics used (cotton and nylon). The prepared functional fabrics with a thickness of 0.1 mm exhibited the EMI shielding performance of 11–15 dB in the frequency range of 8.2–18 GHz [168]. Lightweight and flexible shielding materials are suitable for a wide variety of millimeter-wave shielding applications. Such materials may be prepared using simple and economical methods. In a study, shielding effectiveness (EMSE) of the nanocomposites composed of heat-treated carbon nanofibers (CNF) in a linear low-density polyethylene matrix was assessed. It was observed that the heat treatment (HT) of carbon nanofibers at 2500 °C had remarkably increased the intrinsic transport and graphitic crystallinity, thereby increasing the electromagnetic shielding efficiency of the nanocomposites. The nanocomposites containing 11% vol. % (20% wt.%) of HT exhibited a DC electrical conductivity of 1.0 6 0.1 3 101 S/m, approximately 10 orders of magnitude better than that of the as-received PR-19 CNF nanocomposites. In the frequency range of 30 MHz–1.5 GHz, the nanocomposite containing HT exhibited average EMSE values of approximately 14 ± 2 dB [169]. Epoxy nanocomposites containing 15% of 2 mm thick single-walled carbon nanotube exhibited an EMI shielding performance of 20–30 dB [170]. A higher shielding was gained through the insertion of highly conductive metal particles, such as that in the case of a 4 µm CNT film with approximately 35% wt.% of iron which exhibited a shielding performance of 61–67 dB [171]. In another study, a five-layer CNT film exhibited extraordinary shielding efficiency of 67–78 dB [172]. When the electromagnetic shielding of carboxymethyl cellulose (CMC) and CMC/metal nanoparticles nanofiber mats was investigated, it was observed that the EMI improved in the presence of metal nanoparticles, and was dependent on the concentration and electrical conductivity of the metal nanoparticles [173].

**Table 4:** Shielding performance of some lightweight polymer nanocomposites.

Polymer Nanocomposites	Thickness d(mm)	Shielding (dB)	References
Poly (methyl methacrylate)/Multi-walled carbon nanotubes	0.06	27	[174]
Nitrile butadiene rubber/Fe_3_O_4_	2	80–90	[175]
Poly(vinyl alcohol)/Fe_3_O_4_	4.5	6	[176]
Polyurethane/Multi-walled Carbon nanotubes	0.1–0.2	20–29	[177,178]
Polyacrylate/Multi-walled carbon nanotubes	1.5	25	[179]
Polypropylene/Carbon black	2.8	40	[180]
Polysulfone/Carbon nofiber	1	45	[181]
Polylactide/Graphene	1.5	15	[182]
Polyaniline/Grahene	2.5	45.1	[183]
Polyetherimide/Graphene	2.3	44	[184]
Poly (methyl methacrylate)/Single-walled carbon nanotubes	4.5	40	[185]

The rapid proliferation of electro/electrical technology has rendered it necessary to suppress unwanted electromagnetic radiation for effective system implementation. In order to meet the ever-increasing demand for EMI suppression, the use of PNCs for shielding has increased. PNCs have a low fabrication cost, lighter weight, and relatively simple processability. The interfaces between the embedded nanofillers and the polymer within the PNCs are crucial. It is possible to optimize PNCs for several bands of frequency to encompass all the electronic/electrical systems used in various fields, including the medical, industrial, and military sectors. PNCs are capable of suppressing EMI through the mechanisms of reflection and absorption. Among the various nanomaterials that are embedded into PNCs, magnetic nanomaterials are the most appropriate for using PNCs in electromagnetic shielding. These magnetic nanomaterials include ferrites, carbon-based materials, and flexible dielectric materials. The concentration, shape, and size of the nanofillers have a significant influence on the electromagnetic properties of PNCs. It is important to refine the existing methods and compositions for developing EMI shielding responses. PNCs with larger interfaces could be utilized for various shielding applications. Moreover, these PNCs would produce remarkable polarization effects and dielectric attributes for better EM energy absorption. It would, therefore, be appropriate to investigate the synergy between PNC phases for understanding the possibilities of modification for obtaining materials exhibiting improved performance [186,187].

Although extensive research has already been conducted regarding the use of PNCs in electromagnetic shielding, several challenges remain to be overcome, including the nanofiller distribution in the PNCs, chemical stability of PNCs at extreme conditions, and thermal stability of PNCs at higher temperatures. Additional PNC preparation methods should be used when applying PNCs in electromagnetic shielding to produce better PNCs with a uniform distribution of nanomaterials. In addition, further research is required to develop radiation shielding materials with higher efficiency, better uniform distribution, and chemical and thermal stability. Moreover, the weight, size, and toxicity of PNCs have to be investigated to obtain lighter and thin shields with the least toxicity. Furthermore, using recycled polymer waste materials from industries for developing PNCs could be a promising solution for reducing costs and addressing environmental issues. This approach would be environmentally friendly and economically viable [188].

### 4.4. Food Packaging

**Figure 14:**
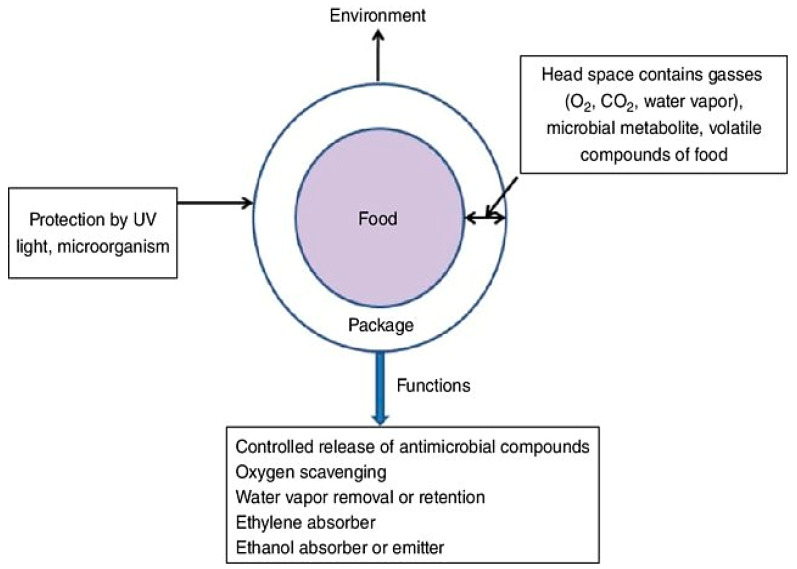
Functions of active packaging to improve self-life of packaged food (taken from [189]).

Food packaging is an icon in the food industry as it plays an important role in modern society. Food packaging facilitates preservation and extension of the shelf life of food products during delivery and until consumption. This is realized through the prevention of unfavorable conditions or factors, such as spoilage microorganisms, chemical contaminants, external forces, moisture, oxygen, light, etc. Figure 14 presents the roles of active packaging in improving the shelf life of food. The food package should prevent microbial contamination, hinder loss or gain of moisture, and act as a barrier against the permeation of oxygen, carbon dioxide, water vapor, and other volatile compounds, including taints and flavors, in addition to the basic properties of packaging materials, such as thermal, optical, and mechanical properties. PNCs represent a novel class of materials that are considered promising candidates as materials for food packaging owing to their outstanding thermal, antimicrobial, mechanical, and barrier properties [189].

Recently, PNCs have been receiving great attention in the food packaging industry. Rahman et al. used a nanocomposite film composed of chitosan and zinc oxide, developed via solution mixing, as the packaging material for extending the shelf life of raw meat [190]. Mahmoodi et al. developed colure biodegradable film nanocomposites composed of PLA, dye, and clay, which exhibited superior light and thermo-mechanical resistance and also provided a barrier to mass transport, rendering these suitable for use as packaging materials [191]. The nanocomposite containing polycaprolactone and zinc oxide nanoparticles exhibited antimicrobial activity against pathogenic *Staphylococcus aureus* and demonstrated great potential as an active food packaging material [192]. Starch nanocomposite films with montmorillonite, prepared through melt processing, are also capable of being used in food packaging. Pirsa et al. developed a novel nanocomposite film based on bacterial cellulose, ZnO NPs, and polypyrrole, which may be useful in antimicrobial active and smart packaging applications [193]. Anothernanocomposite composed of carbon nanotubes and PHBV exhibited enhanced mechanical and thermal properties, facilitating its application in food packaging [194]. Kumar and Gautam reported that starch/ZnO nanocomposite could be used in food packaging applications [195]. Fernández et al. demonstrated that the cellulose/silver nanocomposite exhibited improved antimicrobial activity against spoilage microorganisms during the storage of minimally processed “Piel de Sapo” melon. Moreover, the presence of silver nanoparticles in the nanocomposite resulted in remarkably lower yeast counts and a juicier appearance, in addition to retarding the senescence of melon cuts after ten days of storage [196]. The reinforcement of polycaprolactone with exfoliated graphene oxide produced a nanocomposite that could also be utilized as a packaging material [197]. Cellulose/clay nanocomposite has also demonstrated enhanced mechanical properties, rendering it suitable for use in the packaging of food products [198]. A related study reported the synthesis of a nanocomposite of chitosan with graphene oxide that exhibited outstanding antimicrobial and mechanical behaviors, which render it suitable as a candidate material for the food packaging industry [199].

PNCs have been used in food packaging due to several factors, including their good mechanical and thermal properties, antimicrobial activity, barrier properties, and optical properties. However, certain PNCs may be composed of synthetic polymers or biopolymers incorporated with nanomaterials. It is imperative to limit the use of such PNCs in food packaging, including those that are based on synthetic petroleum-based polymeric films, due to their adverse impacts on human health and also on the environment. On the other hand, the use of PNCs derived from biopolymers could be increased as this exhibit outstanding performance. However, the use of such PNCs for food packaging in industrial fields also remains limited due to several factors, such as their high production cost and unavailability for large-scale production. In order to address these limitations, further research aimed at attaining better mechanical, thermal, and barrier properties along with improved biocompatibility in the PNCs, is required. In addition, the low cost and large scale production of PNC films by avoiding the use of expensive chemicals and rather using economical and natural components would widen the practical application scope of PNCs in various industrial fields [200].

## 5. Conclusions

The present review aimed to describe the current scenario regarding the use of polymer nanocomposites in various fields of applications. As discussed, such applications require producing stable nanoparticles that could be functionalized and exhibit biocompatible properties. The successful development of particles exhibiting specific functional properties for industrial and environmental applications is the greatest challenge for the research community in this field. Polymer nanocomposites hold great importance in the progress of material science. The promising physical and chemical properties of the components of PNCs would enable extending the smart behaviors to material applications. However, much remains to be investigated regarding these systems. Indeed, controlling the challenging and critical variables would provide an even greater impetus for developing these materials and ensuring their potential for upscaling. So far, different preparation techniques have been reported for polymer nanocomposites. However, further investigation is warranted in this regard as well because the intrinsic properties of nanofillers and ensuring their uniform dispersion within the polymer remain a great challenge to date. The preparation method and the types of nanofillers are important factors in determining the type of application a nanocomposite would be suitable for. The polymer nanocomposites may be used in water treatment, sensor, electromagnetic shielding and food packaging. This is attributed to the improved properties exhibited by the polymer nanocomposites compared to the original polymer.

## Data Availability

Not applicable.
